# Prenatal acrylamide exposure results in time-dependent changes in liver function and basal hematological, and oxidative parameters in weaned Wistar rats

**DOI:** 10.1038/s41598-022-19178-5

**Published:** 2022-09-01

**Authors:** E. Tomaszewska, S. Muszyński, I. Świetlicka, D. Wojtysiak, P. Dobrowolski, M. B. Arciszewski, J. Donaldson, A. Czech, M. Hułas-Stasiak, D. Kuc, M. Mielnik-Błaszczak

**Affiliations:** 1 Department of Animal Physiology, Faculty of Veterinary Medicine, University of Life Sciences in Lublin, 12 Akademicka St., 20-950 Lublin, Poland; 2 Department of Biophysics, Faculty of Environmental Biology, University of Life Sciences in Lublin, 13 Akademicka St., 20-950 Lublin, Poland; 3 Department of Animal Genetics, Breeding and Ethology, Faculty of Animal Sciences, University of Agriculture in Kraków, 24/28 Mickiewicza Ave., 30-059 Cracow, Poland; 4 Department of Functional Anatomy and Cytobiology, Faculty of Biology and Biotechnology, Maria Curie-Sklodowska University, Akademicka St. 19, 20-033 Lublin, Poland; 5 Department of Animal Anatomy and Histology, University of Life Sciences in Lublin, 12 Akademicka St., 20-950 Lublin, Poland; 6 School of Physiology, Faculty of Health Sciences, University of the Witwatersrand, 7 York Road, Parktown, Johannesburg, 2193 South Africa; 7 Department of Biochemistry and Toxicology, Faculty of Animal Sciences and Bioeconomy, University of Life Sciences in Lublin, 13 Akademicka St., 20-950 Lublin, Poland; 8 Chair and Department of Developmental Dentistry, Medical University of Lublin, 7 Karmelicka St., 20-081 Lublin, Poland

**Keywords:** Risk factors, Reprogramming, Intrauterine growth

## Abstract

Acrylamide (ACR) is a toxic compound commonly found in fried, baked and heat-processed starchy foods. The current study investigated the time-dependent effects of maternal exposure to non-toxic ACR doses on the oxidative stress, liver function, and basal blood morphology of the rat offspring. Pregnant, Wistar rats were randomly divided into the control group or the groups administrated with ACR (3 mg/kg b.w./day): long exposure for 15 days, medium exposure for 10 days and short exposure for 5 days during pregnancy. Body mass, blood morphology and hematology, serum concentrations of growth hormone, IGF-1, TNF-α, IL-1β, IL-6 and insulin, liver histomorphometry, liver activity of beclin1, LC2B and caspase3, markers of oxidative stress and the activity of antioxidative enzymes in blood serum and the liver were measured in offspring at weaning (postnatal day 21). Even short prenatal exposure to ACR led to oxidative stress and resulted in changes in liver histomorphometry and upregulation of autophagy/apoptosis. However, the most significant changes were observed following the long period of ACR exposure. This study has shown for the first time that ACR is responsible for changes in body mass in a time-dependent manner, which could lead to more serious illnesses like overweight and diabetes later in life.

## Introduction

The chemical reactions which occur during the thermal processing of food products involve carbohydrates, lipids, and amino acids as precursors. One such reaction, the Maillard reaction between the reducing sugars (glucose or fructose) and amino acids, leading to the browning food, is strongly dependent on processing condition and generates toxic compounds like acrylamide (ACR). ACR is present on the surface of the food and is directly linked to the brown color intensity. ACR has been shown to have potential adverse effects on human health and is classified as a group 2A carcinogen for humans and rodents, in accordance with the Commission Regulation (EU) 2017/2158 and the International Agency for Research on Cancer (IARC)^1–3^. The most popular ACR sources include fried potato products, bakery products and even roasted coffee or smoked plums, all of which commonly feature in the diet of pregnant women^4^. The dietary intake of ACR is difficult to assess, since its concentration in many products is unknown, especially that in homemade meals^1,5,6^. Additionally, ACR can be also formed from acrylic acid or acrolein (formed during the cooking of fat), which reacts with asparagine and other amino acids like glutamine, lysine and arginine, and certain protein/peptides, like gluten or carnosine^7–9^. ACR has also been found in thermally processed animal feed, particularly in potato- or wheat-based feeds, with the highest concentrations found in poultry feeds, and it can be transferred to eggs or milk^10–13^. In humans, after oral intake, ACR has also been found in mother`s breast milk^1^. ACR is easily absorbed from the intestine and crosses the placenta^1^. ACR is a known toxin with strong mutagenic, teratogenic and neurotoxic properties, in accordance with the Commission Regulation (EU) 2017/2158 and the International Agency for Research on Cancer (IARC)^3^. Maternal dietary intake of ACR affects the metabolism and general physiology of the embryo/fetus^14–18^. ACR at various doses has resulted in neural tube disturbances, as well as developmental effects, dependent on local or systemic effects^17^. ACR is also considered to be an endocrine-disrupting chemical, which can trigger the reprogramming of development, it impairs physiology and development later in postnatal life^19,20^. ACR-induced maternal/embryonic/fetal toxicity probably occurs through a well-known mechanism involving apoptosis and autophagy, via reactive oxygen species (ROS) induced expression in the mitochondrial apoptosis pathway^21^. Another two possible mechanisms involve ACR-induced interference with the kinesin-related motor proteins, resulting in neurotoxicity and impairments in reproductive function, and the affinity of ACR for sulfhydryl groups on protein, resulting in DNA damage and cancer^22^.

Maternal ACR exposure has been extensively studied in relation to the route of ACR exposure and the wide range of effects, in a dose dependent manner^17,18,23^. A previous study which focused on the embryo/fetal developmental toxicity of ACR in a dose dependent manner, showed that ACR at doses which trigger maternal toxicity, does not affect embryo/fetal development when given during organogenesis^17^. Less attention has been paid to ACR doses which do not trigger maternal toxicity and how they affect offspring in a time-dependent manner. To our knowledge, there are no studies that have investigated the time-dependent effects of maternal exposure to non-toxic ACR doses on the oxidative stress, liver function, and basal blood morphology of their offspring.

The current study aimed to advance on previous reports of animal models of ACR-induced disturbances, focusing on the liver as the main organ system for toxin metabolism, and one of the sites of hemopoiesis during the prenatal period. We hypothesized that fetal ACR exposure can have a profound effect on disease development later in life through the development of oxidative stress. The comparison of three exposure periods allowed us to assess whether a longer duration of ACR-exposure during gestation and into development would result in greater oxidative stress, linked with impairment of liver function and basal blood morphology.

## Results

### Body mass and absolute and relative liver mass

**Figure 1:**
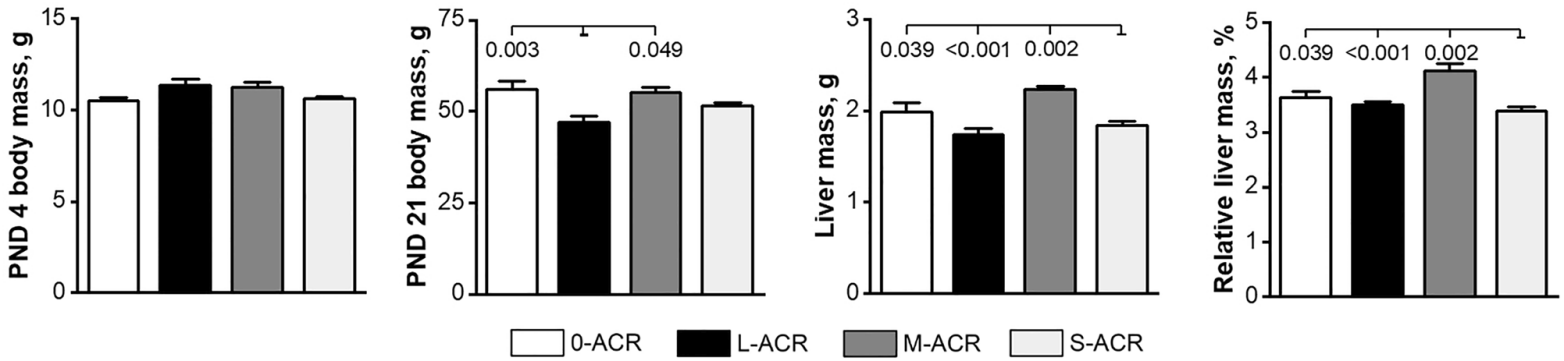
Body mass and absolute and relative liver mass of weaned, Wistar rats not prenatally exposed to ACR (0-ACR) or exposed to ACR from the 6th day (L-ACR), 11th day (M-ACR) or 16th day (S-ACR) of prenatal life up to parturition. Lsmeans and SEM (whiskers) are shown for body mass on PND 4 and PND 21 (final body mass at weaning), as well as liver mass at weaning (PND 21) and relative liver mass at weaning (PND 21). PND–postnatal day. A *p*-value was attributed above plots when two groups exhibit significant differences (*p*-value < 0.05).

The total number of pups and the ratio of male to female pups in each litter was not different across treatment groups. No stillborn animals were observed. The body mass of the offspring on post-natal day (PND) 4 was not different between groups (Fig. 1). At weaning (PND 21), offspring from the L-ACR group weighed significantly less compared to those from the 0-ACR and M-ACR groups. The absolute and relative liver mass of offspring in the M-ACR group was higher compared to that of other groups.

### Blood morphology

The number of WBC in the blood of offspring from the L-ACR group was significantly lower compared to that from other groups (Table 1). Additionally, according to the leucogram, the percentage of lymphocytes and neutrophils in the L-ACR group was significantly higher and lower, respectively, compared to those from offspring in the 0-ACR and S-ACR groups, and did not differ from that noted in the M-ACR group. No other changes in white blood cell parameters were observed.

**Table 1:** Blood hematology parameters of weaned Wistar rats not prenatally exposed to acrylamide (0-ACR) or exposed to acrylamide from the 6th day (L-ACR), 11th day (M-ACR) or 16th day (S-ACR) of prenatal life up to parturition.

	0-ACR	L-ACR	M-ACR	S-ACR	*p*-value
**White blood cell parameters**					
WBC [10^3^/µl]	4.30 ± 0.15^b^	3.60 ± 0.15^a^	4.34 ± 0.14^b^	4.54 ± 0.15^b^	< 0.001
Lymphocytes [%]	74.6 ± 1.2^a^	83.0 ± 2.9^b^	79.7 ± 1.3^ab^	74.9 ± 1.7^a^	0.005
Monocytes [%]	6.04 ± 1.05	4.81 ± 0.91	6.51 ± 0.91	5.98 ± 1.10	0.667
Neutrophiles [%]	19.4 ± 0.8^b^	12.2 ± 2.3^a^	15.5 ± 0.8^ab^	19.0 ± 0.9^b^	< 0.001
**Red blood cells parameters**					
RBC [10^6^/µl]	5.37 ± 0.09^c^	4.57 ± 0.06^a^	5.06 ± 0.07^b^	4.79 ± 0.09^ab^	< 0.001
Hb [mmol/L]	11.74 ± 0.18^c^	9.92 ± 0.16^a^	10.73 ± 0.13^b^	9.87 ± 0.14^a^	< 0.001
HCT [%]	36.4 ± 0.9^b^	30.2 ± 0.5^a^	32.4 ± 0.7^a^	31.7 ± 0.4^a^	< 0.001
MCV [fl]	66.2 ± 1.5	66.3 ± 0.4	64.0 ± 0.6	65.6 ± 0.5	0.674
MCH [pg]	22.2 ± 0.3^b^	22.0 ± 0.2^b^	21.4 ± 0.2^b^	20.4 ± 0.1^a^	< 0.001
MCHC [g/dl]	33.8 ± 0.5^b^	33.3 ± 0.3^b^	33.7 ± 0.4^b^	31.3 ± 0.2^a^	< 0.001
PLT [10^3^/µl]	461 ± 21^c^	208 ± 21^a^	266 ± 20^a^	348 ± 13^b^	< 0.001

Data are presented as Lsmeans ± SEM (standard error of mean). Rows with different superscripts (^a,b,c^) are significantly different (*p*-value < 0.05).

*WBC* total white blood cell number, *RBC* red blood cell number, *Hb* hemoglobin concentration, *HCT* hematocrit, *MCV* mean corpuscular volume, *MCHC* mean corpuscular hemoglobin concentration, *PLT* platelet number.

The number of RBC, Hb content, and the number of PLT in the blood of offspring from the 0-ACR group was higher compared to that in the ACR-exposed groups, among which the lowest number of RBC was noted in the L-ACR group, the highest content of Hb was noted in offspring from M-ACR group and the highest number of PLT in the S-ACR group (Table 1). Hematocrit was significantly lower in all groups exposed to ACR in comparison to that of the 0-ACR group. MCH was lower in offspring from the S-ACR group compared to those in the other groups. MCHC was the lowest in offspring from the S-ACR group, while the MCHC determined in the other groups was similar. No other changes in red blood cell parameters were observed.

### Biochemistry

ALT activity, although highest in offspring from the L-ACR group, was not significantly different from that observed in offspring from the M-ACR group, which in turn was not different from that observed in offspring from the 0-ACR group (Table 2). Offspring from the 0- and S-ACR groups had similar, lower, ALT activity. AST activity was significantly higher in offspring from the L-ACR group compared to the other groups. Offspring from the L-ACR group had the highest ALP activity among all ACR-exposed groups, and was not different from that determined in the 0-ACR group. The concentration of bilirubin was the lowest in offspring from the M-ACR group. The concentration of uric acid was higher in the S-ACR group when compared to that observed in the M-ACR group. The concentration of albumin was higher in offspring from the S-ACR group compared to that from offspring in the other groups. Total protein concentration was higher in offspring from the S- and L-ACR groups compared to that observed in the 0-ACR group, with the total protein concentration in L-ACR group being similar to that in the M-ACR group. Blood glucose concentration was higher in offspring from the M-ACR group compared to all other groups. No other changes in blood parameters were observed.

**Table 2:** Blood biochemical parameters of weaned Wistar rats not prenatally exposed to acrylamide (0-ACR) or exposed to acrylamide from the 6th day (L-ACR), 11th day (M-ACR) or 16th day (S-ACR) of prenatal life up to parturition.

	0-ACR	L-ACR	M-ACR	S-ACR	*p*-value
Fe [µg/dl]	420 ± 10	436 ± 5	440 ± 15	448 ± 8	0.097
ALT [U/L]	27.9 ± 1.0^ab^	36.0 ± 1.1^c^	31.6 ± 1.1^bc^	26.4 ± 0.6^a^	< 0.001
AST [U/L]	155 ± 9^a^	214 ± 11^b^	187 ± 13^a^	169 ± 8^a^	0.003
ALP [U/L]	369 ± 16^ab^	402 ± 13^b^	348 ± 9^a^	334 ± 10^a^	0.002
BR [µmol/L]	7.61 ± 0.54^b^	8.61 ± 0.80^b^	4.17 ± 0.32^a^	6.56 ± 0.18^b^	< 0.001
Urea [mmol/L]	4.83 ± 0.27	5.07 ± 0.33	4.99 ± 0.16	5.06 ± 0.07	0.823
UA [µmol/L]	10.0 ± 0.6^ab^	11.6 ± 0.2^ab^	10.4 ± 0.3^a^	11.8 ± 0.2^b^	0.007
Creatinine [µmol/L]	22.7 ± 1.1	25.1 ± 0.7	24.1 ± 0.7	26.6 ± 1.4	0.059
Albumin [g/L]	25.7 ± 0.5^a^	25.5 ± 0.2^a^	25.3 ± 0.3^a^	28.1 ± 0.7^b^	< 0.001
Total protein [g/L]	44.2 ± 0.3^a^	47.5 ± 0.6^bc^	45.6 ± 1.1^ab^	48.7 ± 0.4^c^	0.001
Glucose [mmol/L]	5.79 ± 0.21^a^	5.62 ± 0.18^a^	7.69 ± 0.19^b^	5.57 ± 0.17^a^	< 0.001

Data are presented as Lsmeans ± SEM (standard error of mean). Rows with different superscripts (^a,b^) are significantly different (*p*-value < 0.05).

*Fe* iron, *ALT* alanine transaminase, *AST* aspartate transaminase, *ALP* total alkaline phosphatase, *BR* bilirubin, *UA* uric acid.

### Hormones

**Figure 2:**
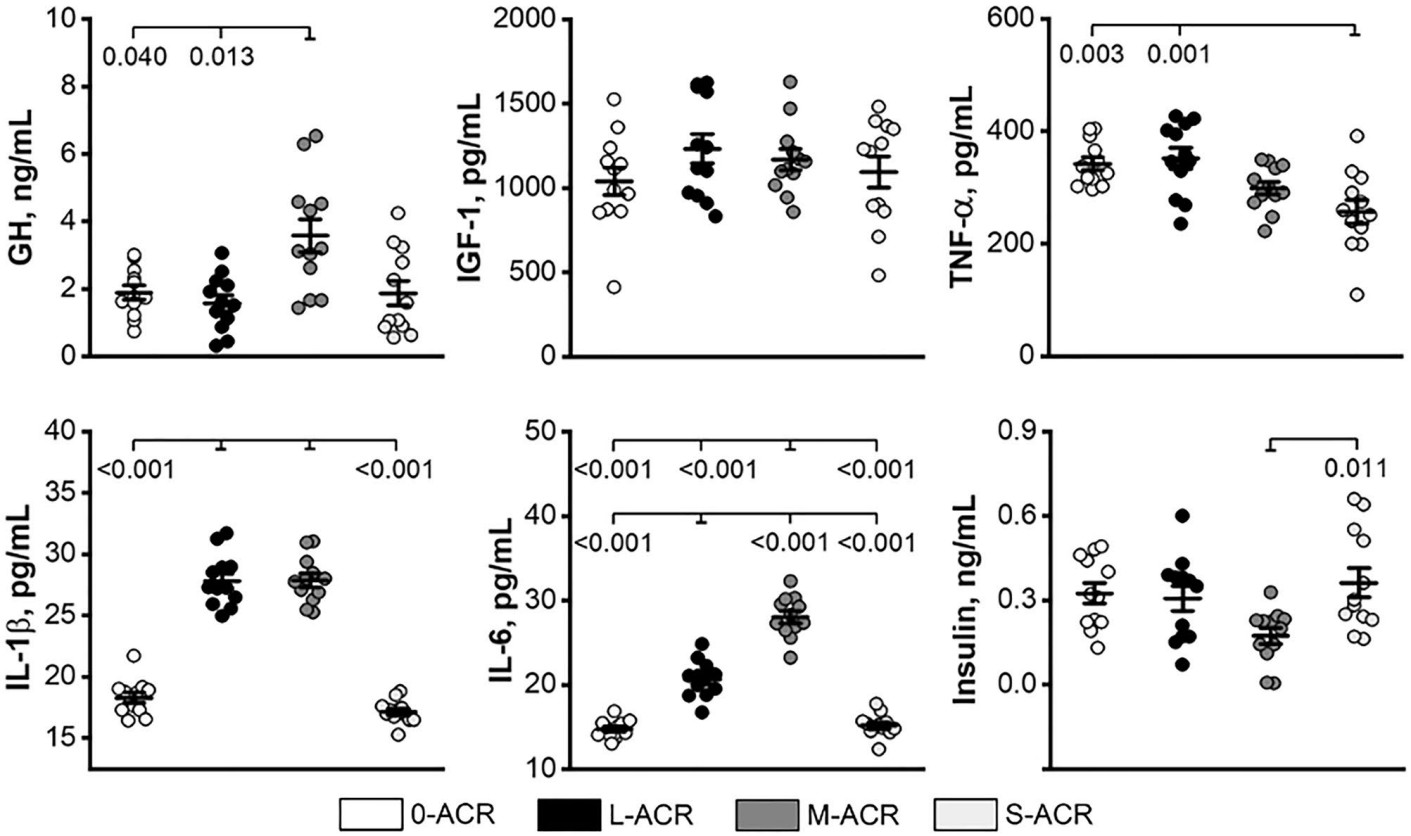
Serum hormone concentrations of weaned Wistar rats not prenatally exposed to ACR (0-ACR) or exposed to ACR from the 6th day (L-ACR), 11th day (M-ACR) or 16th day (S-ACR) day of prenatal life up to parturition. LS means (line) and SEM (whiskers) are shown for growth hormone (GH), insulin like growth factor 1 (IGF-1), tumor necrosis factor alpha (TNF-α), interleukin 1β; (IL-1β), interleukin 6 (IL-6) and insulin. Each data point symbolizes an individual rat. A *p*-value was attributed above plots when two groups exhibit significant differences (*p*-value < 0.05).

The concentration of GH was significantly higher in offspring from the M-ACR group when compared to those from the 0-ACR and L-ACR groups (Fig. 2). Offspring from the S-ACR group had a significantly lower concentration of TNFα compared to those from the 0- ACR and L-ACR groups. The IL-1β concentration was higher in offspring from the L-ACR and M-ACR groups compared to that observed in the 0-ACR and S-ACR groups, which were not different from one another. The concentration of IL-6 was highest in the M-ACR group, lower in the L-ACR group, and the lowest and comparable in offspring from the 0-ACR and S-ACR groups. The concentration of insulin in offspring in the M-ACR group was lower compared to those in the S-ACR group. No changes in IGF-1 concentrations were observed (Fig. 2).

### ROS and antioxidants in blood plasma and liver tissue

**Figure 3:**
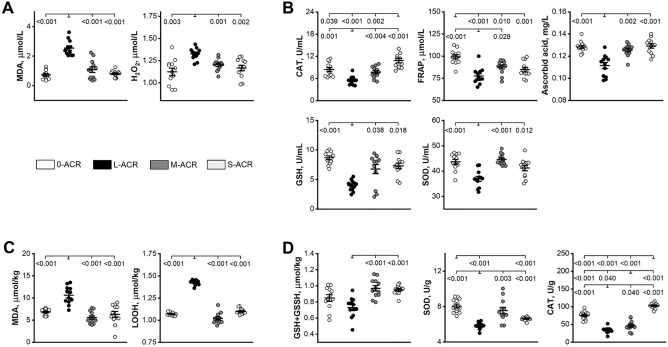
Plasma concentrations of lipid peroxidation products: malondialdehyde (MDA), hydrogen peroxide (H2O2) (**A**), plasma activity of antioxidative enzymes: catalase (CAT), ferric-reducing ability of plasma (FRAP), ascorbic acid, reduced glutathione (GSH) and superoxide dismutase (SOD) (**B**), liver oxidative stress markers, malondialdehyde (MDA) and lipid hydroperoxide (LOOH) (**C**), and liver activity of antioxidative enzymes: total and reduced glutathione (GSH + GSSH), superoxide dismutase (SOD) and catalase (CAT) (**D**) in weaned Wistar rats not prenatally exposed to ACR (0-ACR) or exposed to ACR from the 6th day (L-ACR), 11th day (M-ACR) or 16th day (S-ACR) of prenatal life up to parturition. Data are LS mean values (line) and SEM (whiskers). Each data point symbolizes an individual rat. A *p*-value was attributed above plots when two groups exhibit significant differences (*p*-value < 0.05).

Plasma MDA and hydrogen peroxide concentrations were significantly higher in offspring from the L-ACR group compared to that of offspring from the other groups (Fig. 3A). Significantly lower plasma GSH and SOD activity, as well as ascorbic acid concentration was observed in offspring from the L-ACR group compared to the other groups (Fig. 3B). CAT activity was the lowest in offspring from the L-ACR group, higher in offspring from the 0-ACR and M-ACR groups, and the highest in offspring from the S-ACR group. The highest ferric-reducing ability of plasma (FRAP) was noted in the 0-ACR group. In ACR-exposed groups FRAP was significantly lower in offspring from the L-ACR group compared to that in the M-ACR group.

Hepatic MDA and LOOH concentration, the markers of oxidative stress, were higher in the livers of offspring from the L-ACR group compared to that noted in the other groups (Fig. 3C). Assessment of antioxidant status was performed through the determination SOD, CAT and the sum of reduced glutathione (GSH) and oxidized glutathione (GSSH), both of which exist in healthy cells (Fig. 3D). While the sum of GSH and GSSH in offspring from the 0-ACR group was not different compared to that noted in the groups exposed to ACR, the GSH + GSSH in the L-ACR group was significantly lower when compared to that observed in the M-ACR and S-ACR groups. (Fig. 3D). The lowest hepatic SOD concentration was observed in offspring from the L-ACR group, while it was comparable between offspring from the S-ACR and M-ACR groups, as well as those from the 0-ACR group. The CAT concentration was highest in the livers of offspring from the S-ACR group, lower in the 0-ACR group, and the lowest in the L-ACR and M-ACR groups.

### Liver morphology and immunohistochemistry

**Figure 4:**
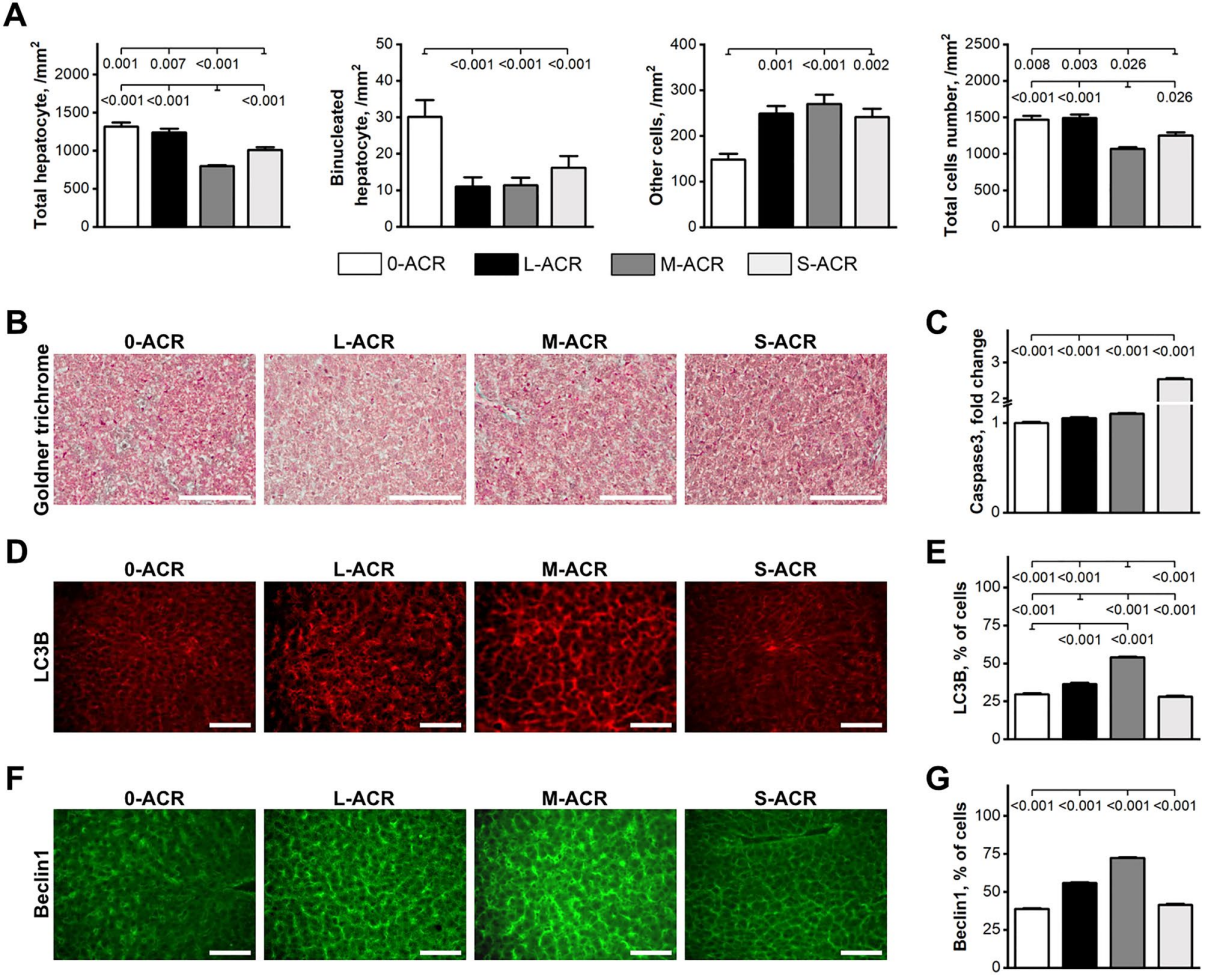
Liver morphology: total hepatocyte, binucleated hepatocyte, other cells, and total cell number malondialdehyde per mm^2^ of liver tissue (**A**), representative images of liver tissue stained with Goldner trichrome (**B**), liver Caspase3 activity (**C**), the expression (**D**) and percentage of immunoreactive cells to autophagy-related protein LC3B (**E**), the expression (**F**) and percentage of immunoreactive cells to Beclin1 protein (**G**) in weaned rats not prenatally exposed to ACR (0-ACR) or exposed to ACR from the 6th day (L-ACR), 11th day (M-ACR) or 16th day (S-ACR) of prenatal life up to parturition. (**B**, **D**, **F**) All scale bars represent 100 µm. (**A**, **C**, **E**, **F**) A *p*-value was attributed above plots when two groups exhibit significant differences (*p*-value < 0.05).

Microscopic assessment of liver histological structure showed no marked differences in the distribution of portal triads and terminal hepatic venules in offspring exposed to ACR compared to those that were not exposed to ACR (0-ACR group) (Fig. 4B). ACR exposure had no effect on the general lobular architecture of the liver tissue, as observed under the microscope on low level magnification. Histological examination of the liver tissue from all groups showed hepatocytes with normal architecture, which were all similar in size and hexagonal in shape, with a nucleus that was more or less centrally located and a homogenous cytoplasm. No hepatocyte ballooning, fibrosis or vacuolization was observed in the hepatic tissue assessed, all of which are usually associated with reduced tissue oxygenation. However, both hepatocyte number, as well as the number of other cells within the liver, were affected by ACR (Fig. 4A). Hepatocyte number was significantly lower in offspring from the M-ACR group, higher in the S-ACR group, and the highest and comparable in offspring from the 0-ACR and L-ACR groups. The number of binucleated hepatocytes and the number of other cells were significantly higher and lower, respectively in the 0-ACR group compared to that observed in ACR-exposed groups. The total number of cells was lowest in offspring from the M-ACR group, higher in the S-ACR group, and the highest in offspring from the 0-ACR and L-ACR groups.

Changes in hepatic autophagy related biomarkers were observed following ACR exposure (Fig. 4D,F). The number of LC3B-positive structures and the area they occupied in the liver was the highest in offspring from the M-ACR group, lower in the L-ACR group, and the lowest and comparable in offspring from the 0-ACR and S-ACR groups (Fig. 4E). The percentage of immunoreactive cells to the autophagy protein Beclin-1 increased significantly in all ACR-exposed groups, with the highest percentage observed in the M-ACR group and the lowest in the S-ACR group (Fig. 4G). All offspring exposed to ACR displayed a significant increase in the fold change in caspase3 activity, with the lowest change observed in offspring from the L-ACR group and the highest fold change observed in offspring from the S-ACR group (Fig. 4C).

## Discussion

Prenatal nutrition plays an important role in the structural development of mammals and has long-term effects evident later in life^15^. The prenatal and neonatal periods are very important for the programming of healthy physical and mental development. Exposure to certain substances/chemicals, via the diet, during these developmentally plastic periods may have detrimental effects later in life. Participants in public health studies based on self-reporting or food frequency questionnaires reported the intake of food products containing ACR by infants, toddlers and other children^1,4,24^. Currently, it is well known that ACR, a processing contaminant, is consumed by humans as part of their daily diet, often unknowingly. A previous study, focused on the embryo/fetal developmental toxicity of ACR in a dose-dependent manner, showed that maternal toxicity starts from a dose of 7.5 mg/kg b.w./day, but this dose does not affect embryo/fetal development during organogenesis^17^. Humans very rarely show symptoms of ACR toxicity due to their chronic ACR consumption at low levels^2,25^. Among the different ACR doses used in rodent studies (1–60 mg/kg b.w./day), the most commonly used dose of 3 mg/kg b.w./day, is not toxic to the dams and suitable to study the developmental/fetal ACR toxicity^2,15,17,18^. Field et al*.*^17^ showed that the maternal ACR dose for which no maternal and fetal adverse side effects were observed is 2.5 mg/kg b.w./day, and 15 mg/kg b.w./ day, respectively. Another study on mice showed that this ACR dose also has no effect on the offspring`s mortality and growth, nor on the animals’ general behavior, as determined four weeks after birth^26^. However, an ACR dose of 3 mg/kg b.w./day triggers slight peripheral neuropathy in female Fischer 344 rats, when administered postnatally for 104 weeks^27^. The ACR dose used in the present study corresponds to a dose of 340 µg/kg b.w. in people. To our knowledge the amount of ACR consumed by people can range from between 30–3,500 µg/kg b.w.^5^. However, a joint FAO/WHO Expert Committee on Food Additives (JECFA) reported ACR consumption of 1 µg/kg b.w. on average and 5.1 µg/kg b.w. (Latin America and Africa) for those that consumed large amounts of ACR^5,19^.

It is well known that dietary ACR crosses the placenta, reduces birth weight and its hemoglobin adducts have been detected in the cord blood of human infants at birth^28,29^. Term body weight is the first indicator of intrauterine development, which determines infant survival and is dependent on the mother’s diet. Dietary ACR increases the risk of development of certain health problems at both early and later stages of life^28^. Some animal studies confirm these observations, while others have presented somewhat contradictory results^15,30^. In the present study, the body weight of offspring on the 4th day after birth was similar in all groups, irrespective of the treatment. Changes in body weight were observed at weaning and were dependent on the period of ACR exposure. Offspring exposed to ACR for the longest period were the smallest. The changes in body mass were linked to changes in GH. Changes in body mass were mirrored by similar changes in liver mass. However, relative liver mass in offspring exposed to ACR for 10 days was significantly increased. It is well known that the liver plays a key role in hematopoiesis during the prenatal period. Hematopoiesis takes place in the yolk sac and paraortic nodes during the embryonic period, after which the liver takes over as the primary site of hematopoiesis. Between the 7th and 8th day of the prenatal period, the pluripotent hemopoietic stem cells are formed in the wall of the yolk sac, from which the spleen colonies and all the primitive myeloid-erythroid progenitors are formed and can be detected in the fetal liver around the 14th day of prenatal life in rodents. The rat fetal liver, like in other mammals, is the primary site of hematopoiesis and can be detected as a distinct organ around the 10th day of prenatal life in rats^31,32^. On about the 17th day of prenatal life, hematopoiesis starts to occur in the bone marrow in the femur and tibia. At the same time the relative liver size decreases. Before the bone marrow takes over as the primary site for hematopoiesis (from the 13.5th of prenatal life), the liver takes up almost the entire abdominal cavity and has up to 8 times more hepatoblasts than normal^33,34^. The rat fetal liver is the site of hematopoiesis between the 11.5th–16.5th day of prenatal life, and there is a close relationship between the developing liver parenchyma and hematopoietic cells, the differentiation of which is strongly influenced by the parenchyma. The fetal liver stops acting as a hematopoietic organ around the 18.5th day of pregnancy, when the parenchymal cells no longer support hematopoietic cell growth^33,34^. At this time the adult type of hemoglobin is detected in the rat fetus^35^.

In the present study, ACR exposure started on the 6th day of the prenatal period, a time at which hematopoiesis occurs in the yolk sac. Offspring from the L-ACR group displayed a decrease in white blood cell number and neutrophils following ACR exposure. ACR exposure, at the time when CFU-S are formed, possibly disturbed the differentiation of pluripotent stem cells into all types of blood cells and thus the repopulation of cells in the spleen and bone marrow. Hashem et al*.*^36^ observed a decrease in neutrophils in rats orally administered 2 mg ACR/kg b.w. for 90 days, along with decreased hematopoietic cells in the hypoplastic bone marrow. Additionally, rats treated with 1 or 2 mg/kg b. w. of ACR have a decreased number of platelets. Decreased platelet formation was observed in the present study irrespective of the period of prenatal ACR exposure. This in turn can lead to problems with coagulation, as was indicated by Hashem et al.^36^. Raju et al*.*^37^ also observed a decrease in platelet number in rats that were administered 10 or 50 mg/kg b.w. of ACR for 8 weeks.

The erythrogram showed that erythropoiesis in all offspring exposed to ACR was disturbed. These results showed that prenatal ACR exposure, even for a very short period, led to a decrease in hemoglobin. The ACR-induced changes in hemoglobin could lead to the development of microcytic anemia, which is commonly related to chronic iron deficiency and hypochromic RBCs. In the present study however, ACR exposure, irrespective of the time period, did not affect plasma iron levels. This result is confirmed by a previous study by Raju et al*.*^37^, in which no changes in iron levels in rats were observed.

**Figure 5:**
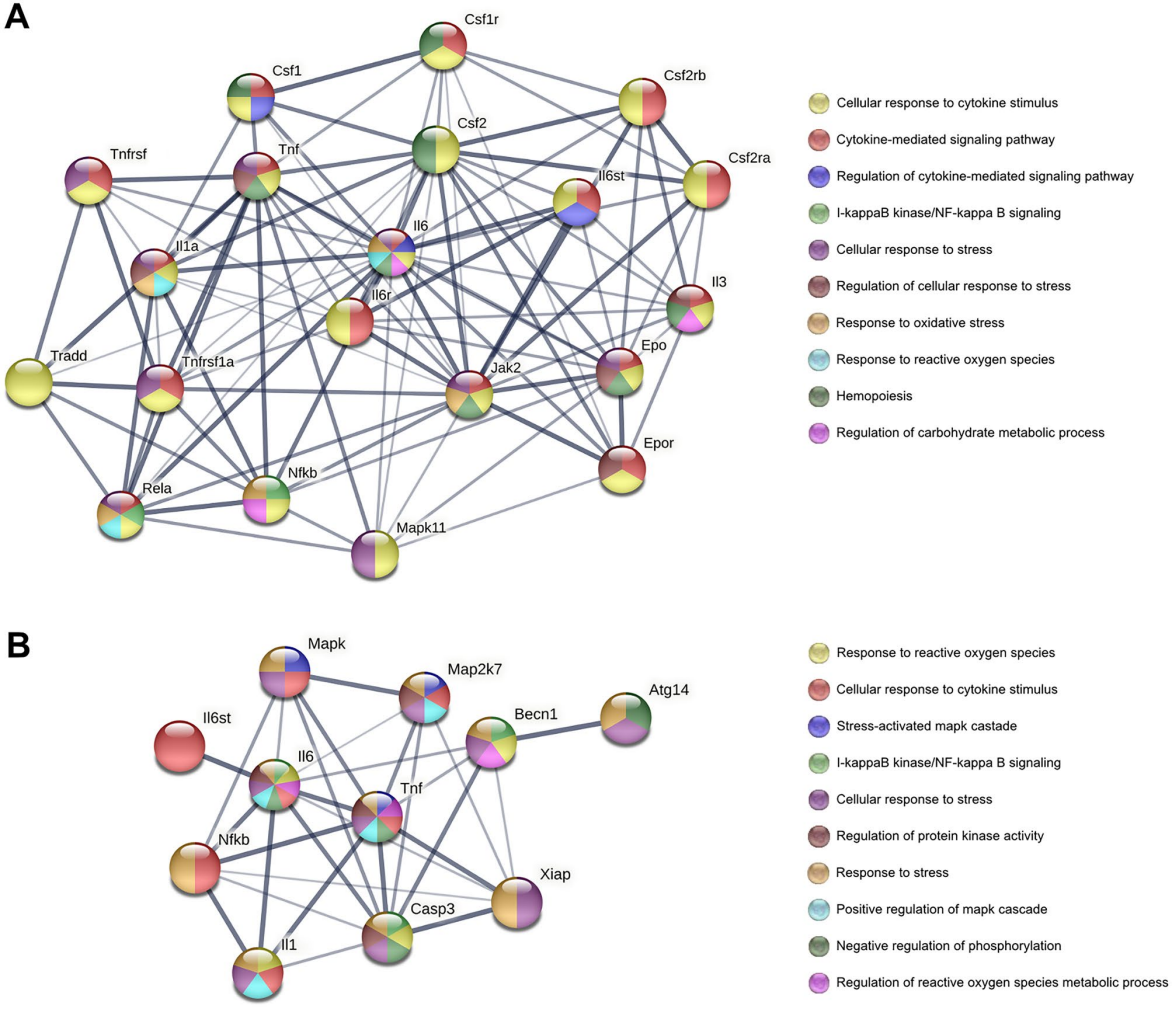
STRING protein–protein interaction diagrams and gene ontology pathways between hematopoietic growth factors and ACR-induced oxidative stress (**A**) and NF-κB pathway components and Becn1 (**B**) (https://string-db.org/, accessed on 12 April 2022). Becn1-Beclin-1; Casp3-Caspase3; SF1- Macrophage colony-stimulating factor 1; CSF2-Granulocyte–macrophage colony-stimulating factor 2; Epo-Erythropoietin; Epor-Receptor for erythropoietin; IL1-Interleukin 1; L3-Interleukin 3; IL6-Interleukin-6; Il6st-Interleukin 6 signal transducer; Map2k7-Dual specificity mitogen-activated protein kinase 7; Mapk-Mitogen-activated protein kinase; Nfkb-Nuclear factor NF-kappa-B; TNF-Tumor necrosis factor; Xiap-E3 ubiquitin-protein ligase.

The prenatal ACR exposure in the present study took place at a time during which liver hematopoiesis appeared, and when the fetal hemoglobin was replaced by the adult type of hemoglobin. The decrease in RBC number observed in all groups in the present study, irrespective of the duration of ACR exposure, could be the result of an additional mechanism involved in the action of ACR, over and above the ACR-induced impairments in bone marrow function. ACR is metabolized into glycidamide in the liver by cytochrome P450, which forms adducts with the sulfhydryl groups in the hemoglobin, via the Michael carbonyl condensation reaction. The formation of hemoglobin adducts could be the reason why low levels of hemoglobin were observed in the RBCs of offspring exposed to ACR^38^. Moreover, it is well known that for the differentiation and growth of hematopoietic stem cells, CFU-S and for the next steps of hematopoiesis, various hematopoietic growth factors (colony-stimulating factors, cytokines, erythropoietin, thrombopoietin) are required. The interaction between all these hematopoietic growth factors, as well as the ACR-induced oxidative stress, should be further investigated (Fig. 5A).

ACR has been shown to induce oxidative stress, and the reaction between ROS (reactive oxygen species) and DNA results in cell destruction; it triggers the inflammatory process, cell apoptosis and autophagy via the mitochondrial apoptosis pathway which is regulated by mitogen-activated protein kinases (MAPK- serine/threonine protein kinases activated by cellular stresses) and the nuclear factor κB (NF-κB) signaling pathway^39,40^. NF-κB is a well-known factor which plays a key role in the transcriptional regulation of genes involved in cell survival and the stress response. ACR exposure results in an increase in NF-κB and the release of inflammatory cytokines (TNF-α, IL-1β, IL-6)^39,40^. The NF-κB and MAPKs signaling pathway is activated by ACR and includes c-Jun N-terminal kinase (JNK) and p38, which in turn affect the proliferation, differentiation, and survival of cells^39^. The p38 MAPK pathway regulates numerous genes which code for a wide range of transcription factors and cytokines including TNF-α, IL-1β and IL-6 (Fig. 5B). In addition, a regulatory role for p38 in the proliferation and differentiation of immune system cells, such as granulocyte–macrophage colony-stimulating factor, erythropoietin and CSF (hematopoiesis growth factor), has been established^41^. Previous studies have reported ACR-induced liver cellular toxicity^19^. It should be emphasized that ACR administered during the prenatal period in the present study affected hematopoiesis, 21 days after cessation of ACR exposure. ACR exposure results in cellular liver toxicity, evident in the basal liver morphology of offspring in the present study. Rats exposed to ACR for 15 days had smaller livers, however the liver size was proportional to their body weight; the livers of offspring exposed to ACR for 10 days were significantly enlarged. The histomorphometrical analysis showed ACR-induced liver damage, where significant changes in hepatocyte number were observed, including that of polyploid cells. The presence of two nuclei is often observed during regenerative processes and is a strategy employed by the hepatocyte to increase its metabolic output. In various liver pathologies, hepatocellular growth shifts to a non-polyploidizing growth pattern^42^. Kupffer cells, the major phagocytic cells found in the liver were also observed. As a result of the ACR-induced hepatic toxicity, Kupffer cells may be activated in order to participate in the repair of injured hepatic parenchyma. The ACR-induced liver injury resulted in an increase in liver marker enzymes, which are commonly released after hepatocyte degradation. The increased activity of AST and ALT was especially evident in the offspring exposed to ACR for the longest period (for 15 days). Further, the increase in autophagy was evident by the degradation of cytosolic proteins and organelles of parenchymal cells. A commonly used marker of autophagy, after the formation of the autophagosome, is the microtubule-associated protein light chain 3 (LC3)^43^. This marker changed in a time-dependent manner in the present study. The highest level of autophagy was observed in rats prenatally exposed to ACR for 10 days, which was confirmed by the level of Beclin-1 observed. Beclin-1 is important for the localization of autophagic proteins to a pre-autophagosomal structure and Beclin-1 regulates the process of autophagy and membrane trafficking involved in several physiological and pathological processes. The relationship between the components of the NF-κB pathway and Beclin-1 is well known^44,45^. NF-κB p65 positively modulates Beclin-1 transcription and autophagy^46^. The levels of Beclin-1 have prognostic significance in liver tumors^47^. The interaction between the components of the NF-κB pathway and Beclin-1 is presented in Fig. 5B. Moreover, caspase-mediated cleavage of Beclin-1 promotes crosstalk between apoptosis and autophagy^45^. The activation of caspase3 has been shown to inhibit autophagy via the cleavage of Beclin-1^48^.

In the present study, caspase3 was investigated by ELISA, while LC3B and Beclin-1 were assessed using immunofluorescence. The level of caspase3, the executor of apoptosis, was significantly increased by ACR exposure in a time-dependent manner. The levels of Beclin-1 and LC3B also changed in a time-dependent manner.

To our knowledge, the present results are consistent with a previous study related to mouse oocytes, which showed that ACR damages mitochondria and induces autophagy/apoptosis, then leads to an increase in the levels of LC3 and Becn1^43^. On the other hand, ACR-induced autophagy (by the mitochondrial apoptosis NF-κB pathway) is inhibited by increased ROS, the accumulation of which results in an increased rate of apoptosis and the secretion of inflammatory factors, and the metabolic changes involving ACR-triggering glycolysis/gluconeogenesis attenuation by the decrease of levels of glycolytic intermediates, reduced rate of the tricarboxylic acid (TCA) cycle, and elevated levels of several amino acid metabolites and lipid metabolites^49^. ACR-induced toxicity is the result of various mechanisms including oxidative stress and an inflammatory process involving increased TNF-α, IL-1β and IL-6 levels^50^. Biochemical analysis has proven that ACR generates ROS, like MDA. Overproduction of ROS results in oxidative protein and lipid production, decreased antioxidants, DNA damage, mitochondrial dysfunction, inflammation, as well as apoptosis^21,50^. Detoxification of ACR and glycinamide occurs through a reaction with GSH, which is excreted in the urine^51^.

The results obtained from the present study showed an increase in MDA and hydrogen peroxide (which freely penetrates the lysosomal membrane and reacts with free iron), and a decrease in GSH, SOD, CAT, FRAP and ascorbic acid, in a time-dependent manner. Additionally, in the rats exposed to ACR, an increase in pro-inflammatory cytokines was observed, which was also time dependent. The significant reduction in all the endogenous antioxidants investigated in the current study was a result of the excessive ACR-induced production of ROS and can result in the initiation of health problems, with developmental origins, later in life. It should be emphasized that in oxidative stress and inflammatory process ACR-triggering is involved NF-κB and MAPKs signaling pathway^40^. These pathways are also key in the ACR-induced liver toxicity^52^.

Although the hepatic expression of NF-κB is not presented in the present study, lipid peroxidation was used as an indicator of ROS-mediated damage to liver cell membranes. The changes observed in MDA and LOOH (a biomarker of the detection and quantification of early-stage lipid peroxidation), were time-dependent. Both markers were increased in rats exposed to ACR for a long period of time prenatally (15 days). These rats also had the highest level of hydrogen peroxide, which causes lipid accumulation and induces lipogenesis in the liver leading to its damage, both of which can in turn also lead to the development of insulin resistance^53^.

Analysis of the antioxidative capacity of the liver also showed time-dependent changes in SOD, CAT and in the sum of reduced (GSH) and oxidized (GSSG) glutathione (both forms are present in undamaged tissue, but the reduced form is the main tissue antioxidant). Other biochemical analyses performed to assess liver function and to detect liver lesions indicated damage to the liver of rats prenatally exposed to ACR, although there were no changes in bilirubin observed (except for the M-ACR rats). On the other hand, a time-dependent increase in uric acid was observed, which is produced in the liver as the final breakdown product of purine. Its elevated serum concentration could indicate liver failure or hematological problems and can lead to acute kidney injury or the development of metabolic syndrome^54^. If creatinine concentrations were increased, this could indicate kidney injury. Moreover, exposure to ACR for a short period of time (5 days) increased total protein. In the case of liver injury, a decrease in albumin and total protein are commonly observed, but the decreased HCT and increased albumin concentration observed, together with the increase in total protein concentration, could point to a serious anemia and dehydration. The significant increase in glucose in the rats exposed to ACR for 10 days (M-ACR group) (this group also had the lowest serum insulin concentration) could suggest the development of insulin dependent diabetes mellitus. Moreover, these rats weighed more at weaning than the other rats exposed to ACR. This could indicate that prenatal ACR exposure can lead to overweight. This observation is consistent with the study by Kadawathagedara et al*.*^55^, who were one of the first to focus on the relationship between prenatal ACR exposure and postnatal growth in people (up to 8 years old), where the link between overweight and high prenatal exposure to ACR is described in line with fetal programming and the developmental origins of health and disease hypotheses.

In our opinion the results obtained from the present study could be of clinical significance, especially since animal studies are an irreplaceable research tool supporting developmental toxicology. Further studies should make use of involve high-throughput sequencing to evaluate the same/different toxic pathways. Moreover, maternal and fetal defense mechanisms against the same substances may differ due to differences in maturity of all mechanisms.

In summary, this is the first study to establish that ACR exposure, confined to the period after conception, has a significant impact on oxidative stress at weaning, with underlying dysfunction relating to hemopoiesis and liver function. Our results are particularly significant as the ACR exposure was at a dose which is not toxic for the mothers. ACR is responsible for the development of oxidative stress in a time-dependent manner, which is considered to be a pathological state preliminary to more serious illnesses like overweight and diabetes later in life.

## Methods

### Ethics approval

All animal procedures were approved by the Local Ethics Committee for Animal Experiments, University of Life Sciences in Lublin, Poland (No 88/2017). The experiment was carried out in compliance with the European Union law (Directive 2010/63/UE, received in Poland by Legislative Decree 266/2015) of the European Parliament and of the Council on the protection of animals used for scientific purposes and following the ARRIVE guidelines for reporting on experiments involving animals.

### Animals

The study was performed at the Experimental Medicine Center of the Medical University of Lublin. The experiment was carried out on offspring delivered by twenty-four female Wistar rats. The pregnant adult dams, with a mean initial body weight of approximately 220 g, were housed individually in cages (55 cm × 33 cm × 20 cm ), under standard laboratory conditions (constant temperature of 22 ± 1◦C, controlled relative humidity within 55 ± 10%, a 12-h day/night cycle, constant room air changes of 15/20 renewals/h) and free access to drinking water and standard laboratory rodents chow (Sniff Spezialdiäten GmbH, Soest, Germany), formulated to meet minimal nutritional requirements of AIN-93 diet^56^.

### Reagents

All chemicals were purchased from Sigma-Aldrich, St. Louis, MO, USA, unless otherwise stated.

### Experimental groups

All dams were weighed daily in order to determine the appropriate ACR dose. The dams were randomly divided into four groups and were dosed with ACR (A8887, Sigma-Aldrich; 3 mg/kg b.w./day, diluted in tap water) or vehicle (tap water) by oral gavage. ACR and vehicle were given at the same volume. The ACR doses were adjusted every other day to account for changes in body weight of the dams. The experimental groups were as follows: the control group, which received no ACR (0-ACR group, n = 6) and the groups exposed to ACR for a period of either 15 days, (long exposure group; L-ACR group, n = 6), 10 days (medium exposure group; M-ACR group, n = 6) or 5 days (short exposure group; S-ACR group, n = 6), from the 6th, 11th or 16th day of pregnancy until parturition, respectively. Starting on the 6th day of pregnancy, all pregnant dams from the L-ACR group, received ACR in tap water, while the other dams from the 0-ACR, M-ACR and S-ACR groups were dosed with vehicle (tap water). The M-ACR group started to receive ACR on the 11th day of pregnancy, while the other two groups were still administered tap water. On the 16th day of pregnancy the L-ACR group started to receive ACR. Thus, ACR was given from the 6th, 11th or 16th day of pregnancy until parturition in the L-ACR, M-ACR and S-ACR groups, respectively. The parturition interrupted the ACR exposure, and during the time until weaning animals were not ACR exposed. After delivery, all newborns were kept with their mothers and were not transferred between dams. On postnatal day 4, the newborns were weighed, after which some of the pups were culled so that each group had eight pups in total (four males and four females where possible) per litter, to normalize rearing. At weaning (postnatal day 21), all offspring were fasted overnight (12 h), weighed, and one female and one male from each dam with the body weight closest to litter average, were anesthetized with an intraperitoneal injection of ketamine + xylazine solution in NaCl (100 mg/kg + 10 mg/kg), according to recommendations for anesthesia and euthanasia of experimental animals and sacrificed by cervical dislocation at the same time in the morning. A total of n = 12 offspring from each group were sacrificed for analyses.

### Liver tissue collection, histomorphometry, caspase3 and immunohistochemical analyses

Livers were taken immediately after euthanasia from each rat, weighed, and then samples from the right lobe were snap frozen in liquid nitrogen and stored at -80 °C until subsequent analyses or fixed in 4% buffered formaldehyde (POCH, Gliwice, Poland; pH 7.0) for 24 h. Formaldehyde-fixed samples were processed into paraffin and stained with Goldner trichrome for morphological examination by light microscopy (CX43, Olympus, Tokyo, Japan). In addition to the histopathological examination, total hepatocyte number, the number of binucleated hepatocytes and total cell number were determined on three separate tissue sections, on at least ten different areas of each section using ImageJ^57^ and CellSens (Olympus, Tokyo, Japan) image analysis software. The measurements were then averaged and expressed as the mean value of the calculated parameters for each rat.

Liquid nitrogen frozen samples were cut at -20 °C in a cryostat into 10-μm thick serial sections. To determine Beclin1 activity, frozen sections were fixed with 4% formaldehyde, as paraformaldehyde, in 0.1 M phosphate buffer (pH 7.4). To determine autophagy-related protein LC3B activity, frozen sections were fixed with methanol. The immunohistochemical stain was performed with anti-Beclin-1 monoclonal antibody (#sc-48341, Santa Cruz Biotechnology, Santa Cruz, Ca, USA; dilution 1:100) and anti-LC3B polyclonal antibody (#2775, Cell Signaling Technology, Danvers, MA, USA; dilution 1:200) as primary antibodies and goat anti-mouse secondary antibody conjugated to Alexa Fluor 488 (#A11001, ThermoFisher Scientific, Walthman, MA, USA; dilution 1:1000) and goat ant-rabbit secondary antibody conjugated to Alexa Fluor 555 (#A21428, ThermoFisher Scientific, Walthman, MA, USA; dilution 4:1000), respectively. Stained sections were examined for percentage of immunoreactive cells on at least ten different areas of each section and then averaged and expressed as the mean value of the calculated parameters for each rat with a fluorescence microscope (Axio Imager A.2, Carl Zeiss, Oberkochen, Germany) and AxioVision (Carl Zeiss, Oberkochen, Germany) image analysis software. All microscopic images collected were examined blindly by an associate who was not aware of the treatment.

Caspase3 activity was determined in liver tissue homogenate using the ready-to-use fluorometric assay kit (#K105, BioVision, Milpitas, CA, USA), according to the manufacturer’s instructions. Fluorescence at 400 nm excitation and 505 nm emission was read in a Fluorescence Microplate Reader FLx800 (BioTek Instruments, Winooski, VT, USA). Caspase3 activity was expressed as the fold-change when comparing the level of caspase3 activity of the ACR-0 group.

### Blood measurements

Whole blood was collected by cardiac puncture into tripotassium salt of ethylenediaminetetraacetic acid (K3EDTA) coated tubes (BD Vacutainer Systems, Plymouth, UK) for hematology, blood plasma determinations, and tubes with silica cloth activator (BD Vacutainer Systems, Plymouth, UK) for blood serum determinations. K3EDTA tubes were centrifuged immediately (1500 × g for 10 min at 4◦C) to separate the plasma. The serum samples were prepared through centrifugation of coagulated blood at 1300 × g for 10 min at 18 °C. Collected serum and plasma were aliquoted into polypropylene tubes and stored at -86 °C until the assays were performed.

The number of white blood cells (WBC), lymphocytes, monocytes, neutrophils, red blood cells (RBC) and platelets (PLT), as well as the hemoglobin concentration (Hb), hematocrit (HCT), mean corpuscular volume (MCV), mean corpuscular hemoglobin (MCH), and mean corpuscular hemoglobin concentration (MCHC) were determined using an automatic hematology analyzer (Advia 2120, Siemens Healthcare, Erlangen, Germany).

Serum was analyzed for alanine transaminase (ALT), aspartate transaminase (AST), total alkaline phosphatase (ALP), bilirubin (BR), uric acid (UA), urea, iron, creatinine, albumin, glucose, and total protein using an automatic biochemistry analyzer (Mindray BS-120, Bio-Medical Electronics, Shenzhen, China) and the respective commercial ready-to-use tests (Alfa Diagnostics, Warsaw, Poland). All analyses were verified with the use of multiparametric control serum (Alfa Diagnostics, Warsaw, Poland).

Serum concentrations of growth hormone (GH, E1997Ra), insulin-like growth factor I (IGF-1, E0709Ra), tumor necrosis factor α (TNF-α, E0764Ra), interleukin 1β (IL-1β, E0119Ra) and interleukin 6 (IL-6, E0135Ra) were determined using commercial rat-specific enzyme-linked immunosorbent assay (ELISA) kits from BT-Lab (Korain Biotech, Shanghai, China). Serum insulin was determined using a commercial rat-specific ELISA kit from Qayee-bio (QY-E11704; Qayee-bio, Shanghai, China). All procedures were performed according to the manufacturers’ protocols. Samples were analyzed in duplicate using a microplate spectrophotometer (Benchmark Plus, Bio-Rad Laboratories, Inc., Hercules, CA, USA). Results were calculated using standard curves created in individual tests.

### Determination of ROS and antioxidant status in blood serum and liver

Lipid peroxidation products—malondialdehyde (MDA) and hydrogen peroxide (H_2_O_2_), as well as the activity of antioxidative enzymes—catalase (CAT), ferric-reducing ability of plasma (FRAP), ascorbic acid, reduced glutathione (GSH) and superoxide dismutase (SOD)—were determined in blood plasma. MDA was determined by the method of Esterbauer & Cheeseman^58^, and H_2_O_2_ was determined according to the Gay & Gebicki^59^ method. The activity of CAT, FRAP, ascorbic acid, GSH and SOD were determined spectrophotometrically according to the methods of Aebi^60^, Benzie & Strain^61^, Omaye, et al*.*^62^, Jollow et al*.*^63^ and Beauchamp & Fridovich^64^, respectively.

Liver tissue was used for measurements of oxidative stress markers, MDA, according to the method of Esterbauer & Cheeseman^58^, and lipid hydroperoxide (LOOH) as described by Gay & Gebicki^59^. The of analysis of the activity of antioxidative enzymes included the assay of the activity of SOD and CAT and total and reduced glutathione (GSH + GSHS) according to Flohé & Günzler^65^.

### Statistical analysis

Data are expressed as the least squares means (lsmeans) and standard error of means (SEM). All statistical procedures were conducted using SPSS Software (v.26, IBM SPSS Statistics, New York, NY, USA). Data were checked for normality (Shapiro–Wilk test) and homogeneity of variance (Levene’s test) and those data that were not normally distributed were log transformed to attain normality. Normally distributed data were analyzed using a one‐way analysis of variance (ANOVA), applying Tukey's honestly significant difference (HSD) and Tamhane's T2 post hoc tests for homoscedastic and heteroscedastic parameters, respectively. Data that could not be transformed to attain normality were analyzed using the non‐parametric Kruskal–Wallis ANOVA with Dunn’s post hoc multiple comparisons test. For all tests, a p-value of less than 0.05 was considered statistically significant.

## Data Availability

The datasets used and analysed during the current study are available from the corresponding author on reasonable request.

## Abbreviations

ACR: Acrylamide
ALT: Alanine transaminase
ALP: Alkaline phosphatase
ANOVA: One‐way analysis of variance
AST: Aspartate transaminase
Becn1: Beclin-1
BR: Bilirubin
b.w.: Body weight
CAT: Catalase
Casp3: Caspase3
CFU: Colony forming unit
CSF: Colony-stimulating factor
CSF2: Granulocyte-macrophage colony-stimulating factor 2
ELISA: Enzyme-linked immunosorbent assay
Epo: Erythropoietin
Epor: Receptor for erythropoietin
Fe: Iron
FRAP: Ferric-reducing ability of plasma
GH: Growth hormone
GSH: Reduced glutathione
GSSG: Oxidized glutathione
Hb: Hemoglobin concentration
HCT: Hematocrit
H_2_O_2_: Hydrogen peroxide
IGF-1: Insulin-like growth factor I
IL-1β: Interleukin 1β
IL-3: Interleukin 3
IL-6: Interleukin 6
Il6st: Interleukin 6 signal transducer
K3EDTA: Tripotassium salt of ethylenediaminetetraacetic acid
LC3: Microtubule-associated protein light chain 3
LC3B: Microtubule-associated protein light chain 3 beta
LOOH: Lipid hydroperoxide
Lsmeans: Least squares means
Mapk: Mitogen-activated protein kinase
Map2k7: Dual specificity mitogen-activated protein kinase 7
MCH: Mean corpuscular hemoglobin
MCHC: Mean corpuscular hemoglobin concentration
MCV: Mean corpuscular volume
MDA: Malondialdehyde
Nfkb: Nuclear factor NF-kappa-B
PND: Post-natal day
RBC: Red blood cells
ROS: Reactive oxygen species
SEM: Standard error of means
SF1: Macrophage colony-stimulating factor 1
SOD: Superoxide dismutase
TCA: Tricarboxylic acid cycle
TNF: Tumor necrosis factor
TNF-α: Tumor necrosis factor α
UA: Uric acid
WBC: White blood cells
PLT: Platelets
Xiap: E3 ubiquitin-protein ligase
